# Insanity—Its Nature and Causes

**Published:** 1889-03

**Authors:** A. N. Denton

**Affiliations:** Austin, Texas; Late Supt. State Lunatic Asylum


DANIEL'S

### TEXAS MEDICAL JOURNAL.

A Representative Organ of the Medical Profession, and an Exponent of Rational Medicine; devoted to the Organization, Advancement and Elevation of the Profession in Texas.

PUBLISHED MONTHLY.—SUBSCRIPTION $2.00 A YEAR.

Vol. IV. AUSTIN, MARCH, 1889. No. 9.

### ORIGINAL ARTICLES.

CONTRIBUTED EXCLUSIVELY TO THIS JOURNAL.

*The Articles in this Department are accepted and published with the understanding that we are not responsible for, nor do we indorse the views and opinions of the writers by so doing.*

### INSANITY—ITS NATURE AND CAUSES.

BY A. N. DENTON, M. D., AUSTIN, TEXAS.

*Late Supt. State Lunatic Asylnm.*

Read at meeting Austin District Medical Society, September 20, 1888, and ordered published in the official JOURNAL.

ACCORDING to promise, I again direct your attention briefly to the subject of insanity. In this paper only the nature and causes will be adverted to. It is scarcely necessary to say that I cannot compass the subject in all its details, so briefly as is here necessary.

Briefly, insanity means a loss of equilibrium or correllation of the mental faculties of man.

The subject is one of profound interest, not only to the metaphysician, but to every human being capable of evolving a single thought. For amid the myriads of mysteries with which man is surrounded, there is none more interesting and perplexing than the existence, nature and destiny of man himself. Man's intense desire to pry into and solve the inscrutable mysteries involved in the problem of life is excusable, if not commendable. But it is not my purpose to discuss the relations existing between the mind and the physical system, except in so far as it may elucidate the subject under discussion. I assume the duality of man's existence, and that the mind, or the soul, if you will, is a substantial, though invisible and intangible entity. This intangible organism permeates and occupies every part of the physical organism, and the latter is the counterpart, or expression of the former. If the theory of the duality of man's existence is excluded, it will be difficult, if not impossible, to account for many of the phenomena observed in heredity. This law of heredity, which is universal, and not confined in its operations to the human race, as might be supposed, is an important factor in the causation of insanity. The truth is, this is the great overshadowing and predisposing cause, whilst the multitude of other causes are but immediate and exciting. At the proper time, these exciting causes will be adverted to briefly, in detail. But how is the tendency to insanity, or other disease, transmitted from parent to children? What part does the physical organism play in the curious phenomena? How much truth is there in the commonly accepted idea that the children inherit, through the medium of blood, the physical peculiarities (whether of strength or weakness,) of their immediate ancestors? Before undertaking to definitely answer these deeply interesting queries, I desire to direct your attention to the well known physiological law of disintegration, or waste and supply of the physical system. All standard works on physiology teach that waste and supply is so rapid and complete as to change every atom composing the human body several times during the average period of a human life. Huxley says, "So constant and universal is this absorption, waste and reproduction, that it may be said with perfect certainty, that there is left in no one of our bodies at the present moment a millionth part of the matter of which they were originally formed.'' He also says, ''Bone once formed does not remain during life, but is constantly disappearing and being replaced in all its parts.'' (Lecture on Origin of Species, p. 28.)

Dr. Flint says, ''It is known that the organic principles of the body, which form the basis of all organs and tissues, are continually undergoing change as a condition of existence; that they do not unite with any substance in definite proportions, but their particles, after a certain period of existence, degenerate into excrementitious substances.'' (Physiology of Man, Vol. I, p. 474.)

Citations from leading authors to this same effect might be indeffinitely extended, but these are deemed sufficient to show the absurdity or impossibility of transmission of character through the blood, or other part of the corporeal structure, from one generation to another. It is impossible for physicists, who hold to the theory of transmission through or by the corporeal system, to successfully meet this argument. It cannot be possible that transmission is thus effected, when it is an universally admitted fact that the body is constantly undergoing change, and is entirely disintegrated and replaced about once in every seven years. Furthermore, if the possibility of transmission of character and disease through and by the corporeal system be admitted, then the question arises, ''How does the father impress his own features and other characteristics upon his children? And why does not the mother exercise an overshadowing influence in the transmission of character and disease to her offspring?'' These queries are suggested by the well known fact that the father furnishes not more than one part in one thousand, if so much, of the physical structure of the child, at time of birth; all the rest is furnished by the mother, and for months arterwards its sole source of supply is from the maternal side. Why, then, I repeat, if this is only a physical problem, does not the mother exercise an overshadowing influence in stamping character and disease upon the offspring? I need not say that such a result is contrary to universal experience and observation. That the father exercises at least an equal power with the mother in stamping the progeny with the characteristics of both, is beyond question. Moreover, many cases have been observed of transmission of the mental and physical characteristics of a first husband, through the mother, to the children of a second husband, after the lapse of many years; and cases are not wanting to prove that the mother has transmitted to her children the features and characteristics of her first love, with whom she had never had sexual congress. The same law has been observed to operate amongst the lower animals.

A curious and striking instance illustrative of this principle is related by Darwin in his work on Origin of the Species: A cow broke from her pen and for a day or two during heat, was in the company of a black and white spotted and horned ox. She was with much difficulty separated from him, and taken to a bull. The calf partook in an amazing degree of the physical characteristics of the ox, being white and black spotted and having horns, although the mother and father and immediate kin on either side were of uniform color and without horns. Here as in the cases of transmission of physical and mental characteristics of a former husband to the children of a second, the medium was unquestionably the mind, as the cow in this case could have no sexual intercourse with the ox, and yet the offspring was indelibly marked with his physical characteristics. Certainly no one will dispute that if such physical peculiarities can be transmitted by mental impression through some vital process, diseases may be bequeathed in the same inscrutable way.

Thus we reach the inevitable conclusion that the transmission of diseases as well as other physical and mental characteristics from generation to generation is not a physical but a mental or vital process. The same theory explains and most satisfactorily the many monstrosities and so called marks, which have been observed and recorded from time immemorial. That these deviations from the usual course of fixed laws, are produced by more or less profound vital impressions upon the child in utero through the mental organism of the mother, there is in my judgment no room to doubt. Reasoning from analogy and exclusion the proposition seems fairly demonstrated. How can we conceive of the possibility of a leaf, a fish or a sample of fruit, being fairly photographed upon the skin of the child in utero, through the physical organism of the mother when we know that the circulation of the fœtus is independent and distinct from that of the mother. Observation teaches that these marks are the result of exceedingly transient causes. A single glance of the mother at some disgusting or revolting object, and a monstrosity is the result, or even the thought of and strong desire for some object causes it to be photographed upon the skin of the fœtus. That these phenomena are not the result of physical process is certain, and if this hypothesis is excluded, as it must be by every thinking creature, then they are nndoubtedly the result of mental impressions; and that the organism, through and by which these results are produced is a substantial though invisible and intangible entity, is no less certain.

The theory that the transmission of mental and physical characteristics from one generation to another is accomplished through the mental organism is in harmony with what knowledge we possess as to the nature, formation and development of the ovum.

Physiologists have not yet discovered even with the aid of the microscope any difference between the ultimate or primordial cell of the human ovule and that of other living creatures. Physically, to all appearances, they are identical, and it is quite probable that they are alike in every physical respect. This being conceded, why is the human ovule after the sexual contact more likely to develop into the shape and with attributes of a human being than into a chicken or other specimen of lower animals? What philosopher is able to give a satisfactory or even rational answer to this question, from a purely physical standpoint? No reasoning which does not recognize the duality of every living creature, and the substantial nature of a living, vital organism, and the counterpart of the physical, can satisfactorily account for the differential development of the vitalized ovule during the early period of its existence into the different forms of living creatures.

This power of specific development from the primordial cell into the different forms of animal life is a vital impression, written, so to speak, upon the vitalized germ at the moment of conception, and the law of its future development into a specific form is from that moment fixed.

If it be objected that we are ignorant of the nature of this ideal organism or the modus operandi of its action, I reply, so we are of electricity in all its forms, but is there any one so incredulous as to doubt the existence of this agent, because it cannot be seen or felt?

I have thus somewhat in detail, indicated my views on the subject of heredity, and the transmission of characteristics and diseases from one generation to another. This has been done, not on account of its special applicability to the subject under discussion, but for the purpose of correcting, as far as possible, the erroneous, but commonly accepted theory of physical transmission. When we consider, however, the widespread or almost universality of heredity, as a cause of insanity, these details will not be considered inappropriate. I have already said that heredity is the great and indeed the only predisposing cause of insanity. Of its widely extended influence as a cause there is no doubt, but strange to say, notwithstanding the overwhelming proof, and the daily observation and experience of alienists everywhere, confirmatory of this universal law of heredity, like vaccination, and on no better grounds, it has its opponents who positively deny either its influence or existence, as a cause of insanity or any other bodily or mental defect. Perhaps the most notable and distinguished of these dissentients was the late Dr. Jno. P. Gray, of Utica, N. Y., whose testimony before the criminal court in Washington City in the memorable case of Chas. Guiteau, the assassin, excited the most extended and generally unfavorable criticism. That there should be found so distinguished an alienist as Dr. Gray, who should deny without qualification, as the writer has heard from his own lips, the very existence of heredity as a cause of insanity, is certainly a matter of profound astonishment to a great majority of the profession. The contrary is so well established, and almost universally admitted, that I should deem it a waste of time to undertake to adduce aguments to sustain it.

The influence of heredity as a cause of insanity is especially great in the United States, as compared with other nationalities of the civilized world, as will be explained when we come to consider the prevention of insanity. While it is unquestionably true that in a great majority of cases oi idiopathic insanity, upon careful investigation, heredity will be found to have been the first or primary cause, the idea is not intended to be conveyed, that cases do not arise independent of this cause. For such instance, however, when the system is uncontaminated by hereditary taint, the immediate cause must be more profound and overwhelming, and the prospect for ultimate and permanent recovery is more favorable.

The exciting or immediate causes of insanity, are numerous, and seem to be constantly increasing with the advancing civilization of the age. The great diversity of pursuits, together with the excitement and anxiety to grow rich and supply many artificial wants and injurious luxuries incident to advanced civilization, are not useful adjuncts to the mental integrity of man. The brain being the throne of intelligence, the fountain of thought, it scarcely need be said that upon its healthfulness, symmetry and integrity depends normal mental action. As a rule, any great deviation in size or shape of the brain from a normal standard, will be attended with corresponding mental defects, and whether such asymmetry or other defect be congenital or acquired, the corresponding mental obliquity or asymmetry is the same. Or in other words, it is the physically defective who are most liable to insanity, and as a rule it is this class who furnish the bulk of the population that fill our public asylums. The prevalent idea that the best developed and wisest are most liable to insanity is extremely erroreous. Any one entertaining such views may be quickly cured of his error by walking through the wards of one of our public asylums, and attentively observing the physical aspect of the inmates. He will be impressed with the diminutive heads, and misshapen bodies as well as the general asymmetry of the great majority of the population.

Before passing to the consideration of special or immediate causes of insanity it will be of interest to briefly glance at the brain in its normal condition. The student of psychology, in order to be able to judge and comprehend the mind in its diseased condition, must also acquire a knowledge of the same in a state of health. He must have some knowledge of the physical structures which combine to form the cerebrum, and contribute to its healthy working, and it will be interesting and useful to trace the growth of these structures from infancy to mature age. In doing so he will observe that the growth and development of the mental faculties are as gradual as those of the physical structure, but he will also observe, that in some the mental development is more rapid than in others and that it is liable to be arrested in its development or stunted in its growth, by interference in any way with physical growth and development, or it may be impeded or stopped, by the destruction or imperfection of organs, as in blindness or deafness. The brain is made of nerve cells and nerve fibres, of a substance in which the cells are imbedded, called neuraglia, of blood vessels and lymphatics. The cells are gathered into centre or convolutions, and are the seat of intelligence. The fibres are the conducting media, which communicate with or lead from the convolutions to organs of special sense to the sensary ganglia, and to the convolutions of the other hemisphere.

Modern science is endeavoring to throw additional light on the workings of this mysterious laboratory of human thought. At present we only know that in the gray cellular structure of these convolutions, thought and reason reside and judgment sits enthroned. Since this discovery, which now seems settled, it has naturally occurred to scientists that since the gray matter of the convolutions is the only seat of reason and intelligence, mental power should be in proportion to the amount of the gray matter, or extent and depth of the convolutions. It will require further experiment and observation to sustain this inference, but experience, thus far, seems to point to its confirmation. My own experience and observation, which extends over a considerable field, lead me to the endorsement of the theory. For many years I have, from experience, been led to believe that the injury of the convolutions, or the destruction of the gray cellular matter, from whatever source, is more apt to produce mental disturbance than a similar injury or destruction of the white, or fibrous matter. Further experiment and observation on an extended scale is needed to fully establish this hypothesis, but the probability of its truth is strongly impressed upon the mind of the author of this paper. Concerning the physiology of the blood supply to the brain, there is also some doubt and speculation. We know that the internal carotid and vertebral arteries, forming the circle of Willis, is the principle source of supply. The terminations of these vessels ramify in the pia mater, and thence in the brain substance, the gray, or cellular structure receiving much the larger supply. To the student of psychology, however, the most interesting subject connected with the blood supply to the brain is the vaso-motor system of nerves, which contracts and regulates the blood supply. A strong impression exists at this time in the minds of many alienists, that insanity, in its early or acute stages, consists in little else than increased blood supply to the gray cellular structure of the convolutions. Upon what does this condition depend? Evidently upon some failure of function of the vaso-motor system. But as to the causes of this failure, in a great majority of cases we are yet in the dark. The truth is, that much is yet to be learned before the pathology of insanity can be certainly stated.

By some observers the lymphatics are believed to play an important part as a factor in the production of insanity. The supposition is that these important vessels in certain cases fail to remove the waste of disintegration of the tissues, and thus the brain becomes burdened and poisoned with excrementitious matter, and its healthful equilibrium is destroyed. This failure of functions on the part of the lymphatics may be brought about in many ways: by an occlusion of the caliber of these vessels; by syphilitic deposits, or from other causes. Even the stroma or neuraglia of the brain which seems to be without function, except to serve as a kind of frame work to sustain the cellular structure, is charged by some with an important agency in the production of insanity. Observers have noticed the increase of this structure in diseased brains, which probably only points to an hypertrophy of a lower tissue at the expense of a higher. Passing to the functions of the brain, we reach the most interesting field for investigation, but unfortunately man with all his enthusiasm and thirst for knowledge, has only been able to penetrate to a very limited depth, in this direction, beyond which arises a barrier that seems to be impassable. The exact nature of the relation existing between mind and the physical system is unknown and probably not to be discovered through the unaided faculties of man. Mind implies feeling, consciousness, sensation. But as the student of mind has no consciousness or feeling except his own, how is he to investigate, and study the minds of others? Certainly the subjective examination of ourselves is as inadequate to explain the phenomena of diseased mind, as objective observation is to explain the nature of mind in general. By using the subjective method of observation as our key, we may then objectively examine the mental characteristics of others, and thus in a measure we may acquaint ourselves with the growth and development, disease and decay of mind and form approximately at least, a correct opinion as to mental deviations from a normal standard.

We learn by observation that muscular movements brought about by nerve functions in infancy is automatic or instinctive.

These movements are not effected in obedience to the will and directed by a higher intelligence, which comes by future progress and development in after years. These primary automatic movements are only in a very slight degree attended by consciousness. But in harmony with the growth of the body, the mental organism is slowly developed; and memory that great store-house of the mind, begins to be manifested, and ideas are formed. By memory we are enabled to store away the experiences of the past for future use; not only our own experience but that of others. Here I again record my dissent from the theory taught by leading physiologists, as to the methods by which the experiences of the past are retained for future use.

It is claimed and taught, to be a purely physical process; that these incidents or experiences, are stored away in the various centres, and that by means of nerve fibres, they are united into groups, forming an exceedingly complex system.

Without wearying you by lengthy quotations, I may state in brief that the clear inference is, from an attentive perusal of the standard works on physiology, that the mental impressions, of whatever nature, which constitute memory, is by some molecular, or other species of change, stamped upon the physical structure of the brain, and retained for an indefinite time for future use.

It is a well known fact that many of these impressions are retained, and that vividly, and in a perfect state of preservation, during almost the entire period of a long human life. It is therefore manifest that if these impressions were purely physical, by any species of molecular change in the brain centers, however bright the picture may be in the beginning, it must inevitably be blotted out, utterly expunged, in the course of a few years. No sophistry of reasoning can avoid this result, if the doctrine of disintegration, waste and supply of the physical system is true. This theory has already been adverted to in the earlier pages of this paper, and need not be further elaborated here. Suffice to say, that the physical theory or process is totally inadequate to account for these phenomena of mental retention of past experiences for future use, which we call memory, and this hypothesis being excluded, we are driven to the only other rational explanation, that these impressions are made upon and retained by an intangible but substantial mental organism. I shall not attempt, nor claim the ability to explain the manner or method by which these impressions are made and retained. The facts, however, remain, and cannot be successfully controverted. Like many of the phenomena incident to the workings of other intangible substances of nature, such as electricity, light, et cætera, we are unable to give a satisfactory explanation as to the method of their production; but of results we can form a correct opinion. It must be admitted that a normal mental condition cannot exist without a corresponding healthful condition of the physical system, and especially of the brain. Striking illustrations of this principle are observed in certain pathological conditions of the brain in which the memory is completely expunged, as in case of softening at certain points, in the gray matter of the convolutions. In other cases a variety of amnesia is produced, which consists in a curious inordination of the faculties of the memory. In this condition the patient frequently substitutes wrong words for those he wishes to use. For instance, if he wishes a knife, he asks for a hat or other object. In severe cases of this kind, the patient is incapable of framing a sentence expressing the idea which he wishes to convey. For the growth and healthful development of the mind, all the conditions of physical health are necessary. If there is any physical defect at birth or subsequent injury involving an arrest of physical development, the mind suffers in a corresponding degree, and idiocy or insanity may be the result. In such cases there is no record, or perhaps there is only an imperfect record, of the experiences of life, and therefore an inability to learn, or use to advantage the imperfect impressions of past experiences. If growth and development of the physical system proceeds normally to mature age, a normal mind is the result. Supposing this result to have been attained, what are the special conditions necessary to the production of disordered mind, or insanity? The answer to this query involves the consideration of the special or immediate causes of insanity, which will be adverted to on a future occasion.

